# Rationale and design of a prospective, randomised study of retrograde application of bone marrow aspirate concentrate (BMAC) through coronary sinus in patients with congestive heart failure of ischemic etiology (the RETRO study)

**DOI:** 10.1186/s12872-019-1011-9

**Published:** 2019-01-31

**Authors:** L. Pleva, P. Kukla, K. Vítková, V. Procházka

**Affiliations:** 1 Department of Cardiovascular Diseases, University Hospital Ostrava, tr. 17. listopadu 1790, 708 52 Ostrava 8, Czech Republic; 2 Department of Science and Research, University Hospital Ostrava, Ostrava, Czech Republic; 3 RDG Institute, University Hospital Ostrava, Ostrava, Czech Republic

## Abstract

**Background:**

Heart failure (HF) is a major chronic illness and results in high morbidity and mortality. The most frequent cause of HF with reduced ejection fraction (HFREF) is coronary artery disease (CAD). Although revascularisation of ischemic myocardium lead to improvements in myocardial contractility and systolic function, it cannnot restore the viability of the already necrotic myocardium.

**Methods/design:**

The aim of our prospective randomised study is to assess the efficacy of the retrograde application of non-selected bone marrow autologous cells concentrate (BMAC) in patients with HFREF of ischemic aetiology. The evaluated preparation is concentrated BMAC, obtained using *Harvest SmartPReP2* (Harvest Technologies, Plymouth, MA, USA). The study population will be a total of 40 patients with established CAD, systolic dysfunction with LV EF of ≤40% and HF in the NYHA class 3. Patients have been on standard HF therapy for 3 months and in a stabilised state for at least 1 month, before enrolling in the clinical study. Patients will be randomised 1:1 to either retrograde BMAC administration via coronary sinus or standard HF therapy. The primary end-points (left ventricular end-systolic and end-diastolic diameters [LVESd/EDd] and volumes [LVESV/EDV] and left ventricular ejection fraction [LV EF]) will be assessed by magnetic resonance imaging. The follow-up period will be 12 month.

**Discussion:**

The application of bone marrow stem cells into affected areas of the myocardium seems to be a promising treatment of ischemic cardiomyopathy.

The Harvest BMAC contains the entire population of nuclear cells from bone marrow aspirates together with platelets. The presence of both platelets and additional granulocytes can have a positive effect on the neovascularisation potential of the resulting concentrate. Our assumption is that retrograde administration on non-selected BMAC via coronary sinus, due to the content of platelets and growth factors, might improve left ventricular function and parameters compared to standard HF therapy. Furthermore, it will be associated with improved exercise tolerance in the six-minute corridor walk test and an improvement in the life quality of patients without increasing the incidence of severe ventricular arrythmias.

**Trial registration:**

(ClinicalTrials.gov; https://clinicaltrials.gov; NCT03372954).

## Background

Heart failure (HF) is a major chronic illness and results in high morbidity and mortality; for instance, the mean mortality rate of severe heart failure in the NYHA IV class is 40–50% per year [1]. The most frequent cause of left ventricular systolic dysfunction and the development of HF with reduced ejection fraction (HFREF) in developed countries is coronary artery disease (CAD). Although percutaneous coronary angioplasty and surgical revascularisation of ischemic myocardium lead to improvements in angina pectoris, myocardial contractility and systolic function, none of these methods can restore the viability of the already necrotic myocardium [1].

Substitution of impaired myocytes in affected areas of the myocardium could stimulate cardiomyocytes regeneration, support the neovascularisation and thus prevent remodelling of the left ventricle. Primary pluripotent progenitor cells in bone marrow are able to disperse into functional vascular tissue, which has led to great interest in their use in the treatment of acute myocardial infarction (MI), left ventricular systolic dysfunction and HF [2].

As preclinical data prove, bone marrow autologous cells concentrate (BMAC) are able to separate in vascular structures and with the aid of paracrine mechanisms can improve the function of existing cardiomyocytes or angiogenesis [2, 3]. Studies have already shown that the administration of BMAC leads to improved myocardial perfusion and left ventricular function with minimal adverse effects, and it is therefore safe and offers potential clinical benefits [4] and contrary to skeletal myoblasts, there is no evidence of increase in malignant arrhythmias [1–3].

### Aim

The aim of this prospective randomised study is to assess the efficacy of the retrograde application of non-selected BMAC in patients with HFREF of ischemic aetiology. The evaluated preparation is concentrated BMAC, obtained using *Harvest SmartPReP2* (Harvest Technologies, Plymouth, MA, USA).

Our assumption is that non-selected BMAC administrations will lead to improvements in the left ventricular ejection fraction (LV EF), the left ventricular end-systolic and end-diastolic diameters and volumes (LVESd/EDd and LVESV/EDV) compared to standard HF therapy. Furthermore, it will be associated with improved exercise tolerance in the six-minute corridor walk test, a decrease in NYHA and CCS classes and an improvement in the life quality (QoL) of patients. At the same time, this therapy will not increase the incidence of severe ventricular arrythmias detected by 24-h ECG Holter monitoring.

## Methods/design

The study population will be a total of 40 patients with established CAD and left ventricular systolic dysfunction with an EF of ≤40% and HF in the NYHA class 3. CAD will be demonstrated based on previous coronary angiography (myocardial infarction > 6 months, presence of significant coronary stenosis, previosu revascularisation) or Tc SPECT (perfusion defects). The patient follow-up duration will be 12 months.

Patients have been on standard HF therapy for 3 months and in a stabilised state for at least 1 month, before enrolling in the clinical study. A list of inclusion and exclusion criteria is provided in Table 1.

**Table 1:** Inclusion and exclusion criteria

Inclusion criteria:	
• Patients with chronic heart failure and left ventricular ejection fraction ≤40% with coronary artery disease and with symptoms of heart failure in the NYHA class 3 on standard heart failure therapy for 3 months and in a stabilised state for at least 1 month,	
• Age 40–80 years	
• Informed, written consent by the patient	
• Ability to comply fully with the study protocol	
• Negative pregnancy test (and effective contraception) in women with childbearing potential	
Exclusion criteria:	
• Previous bone marrow disease (especially myelodysplastic syndrome or non-Hodgkin’s lymphoma)	
• Repeated acute coronary syndrome <1 month	
• CRT device with LV lead in CS	
• Active infection or ATB treatment <1 week	
• Previous malignant ventricular arrhythmias without ICD implantation	
• Anemia (HTC ≤ 28%), leukocytosis (≥14.000/mm^3^) or thrombocytopenia (≤50.000/mm^3^)	
• Previous bleeding diathesis	
• Need for hematopoietic growth factor treatment (e.g. erythropoetin, G-CSF)	
• Impossibility of aspiration 240 ml of bone marrow	
• Hepathopathy or cirrhosis (bilirubin, ALT or AST ≥2,5x ULN)	
• Chronic kidney disease >3b stage (eGFR <45 ml/min/1.73m^2^)	
• Uncontrolled hypertension	
• Need for high dose (> 7.5 mg/day) corticotherapy within the next 6 months	
• Inability to stop anticoagulation therapy (> 72 h) before bone marrow aspiration	
• Known malignancies requiring actino or chemotherapy, or previous actinotherapy	
• Patients with a BMI > 40	
• Known allergy to contrast agents	
• Other comorbidities with a life expectancy of 6 months	

Patients will be randomised 1:1 to one of the following treatment methods: either retrograde BMAC administration via coronary sinus or the control group with standard HF therapy. Patients randomised to the control group will not undergo bone marrow collection, due to ethical reasons. Only standard right heart catheterisation will be performed as sham procedure.

The primary objectives of the clinical study are to improve the LV EF and reduce the LVESd/EDd and LVESV/EDV assessed by magnetic resonance imaging (MRI), and to improve exercise tolerance in the six-minute corridor walk test.

The secondary objectives include identifying the occurrence of severe ventricular arrhythmias according to 24-h ECG Holter monitoring, improving on the NYHA and CCS classes, reducing hospitalisations due to decompenzation of HF, assessing the impact on QoL of patients using the Kanas City Cardiomyopathy Questionare (KCCQ-12) and ensuring the safety of BMAC retrograde administration via coronary sinus by evaluating all adverse events (AE).

All patients will continue on the standard HF treatment during the clinical follow-up (ACE inhibitor/ATII blockers, beta-blockers, MRA, diuretics) and medication necessary for secondary CAD prevention (ASA/clopidogrel, statins). The monitored parameters will be measured echocardiographically in ICD-implanted patients not able to undergo MRI. Patient with cardiac resynchronization therapy (CRT) device (or CRT-ICD) with LV lead placed in CS will not be included. We also not include patients with known contrast allergy.

Written informed consent was obtained from each patient before enrollment in the study. The study protocol complied with the Declaration of Helsinki and was approved by the Ethics Committee of University Hospital Ostrava, Czech Republic. The study was registered at ClinicalTrials.gov (https://clinicaltrials.gov; NCT03372954).

### The course of clinical study

**Fig. 1:**
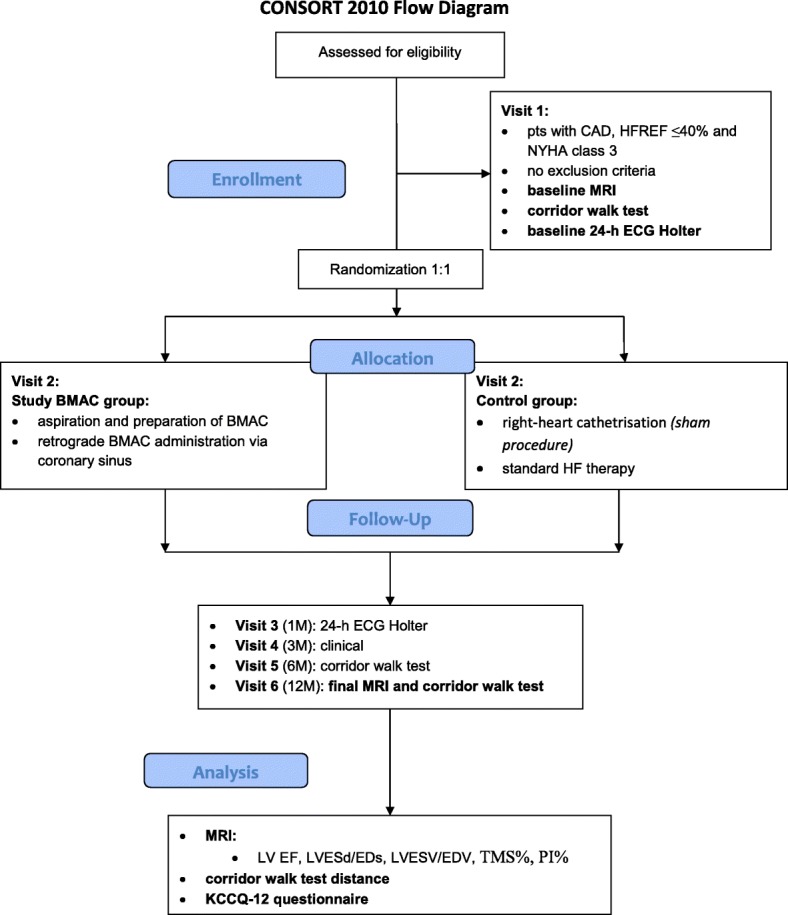
CONSORT study flow diagram

The course of the clinical study is illustrated in Fig. 1 and Table 2.

**Table 2:** SPIRIT overview of study visits and procedures

	Study Period					
	Enrolment	Allocation	Post-allocation			Close-out
	Visit 1	Visit 2	V3	V4	V5	Visit 6
Timepoint	-1 month	0	1 M	3 M	6 M	12 month
Enrolment						
Eligibility screen	X					
Informed consent	X					
Biochemistry	X	X		X	X	X
Blood count	X	X		X	X	X
NT-pro BNP	X	X		X	X	X
CK-MB, troponin I	X	X		X	X	X
ECG	X	X	X	X	X	X
Allocation		X				
Interventions						
BMAC application		X				
Standard HF therapy		X				
Assessments						
MRI/ECHO	X					X
LVESd/LVEDd	X					X
LVESV/LVEDV	X					X
LV EF	X					X
TMS%; PI%	X					X
Corridor walk test	X				X	X
24-h ECG Holter	X		X			
Kanas City Cardiomyopathy Questionare	X				X	X

### Aspiration and preparation of bone marrow autologous cells concentrate (BMAC)

Bone marrow aspiration will be performed in local anesthesia with lidocaine and analgosedation with propofol or midazolam using a Yamshidi aspiration needle, and 240 ml of bone marrow will be aspirated and collected in an ACD-A transfer bag. A sample of 5 ml will be taken to assess the baseline count of nuclear cells.

The aspirate will be transferred to centrifuge bottles to produce BMAC, using the *Harvest SmartPReP2* instrument. Next, 2 ml of 8.4% bicarbonate will be added to the obtained concentrate, to adjust pH, and 18 ml of the patient’s plasma – the original 240 ml of aspirate will thus produce a total of 60 ml of BMAC. Quantitative and qualitative analyses of 5 ml of bone marrow aspirate (BMA) and 5 ml of BMAC will be perormed to assess the cellular composition and content of CD43+ cell. The remaining part of the concentrate will be transferred to opaque 10 ml syringes for application.

### Retrograde administration procedure

Coronary sinus cannulation will be performed via the femoral vein under standard conditions. The diameter of the coronary sinus will be measured using contrast medium injection. An *Advance CS* (Cook, Bloomington, IN, USA) balloon catheter of appropriate size will then be inserted onto a 0.035″ guide wire and inflated (at a maximum pressure of 2 atm), and a small amount of contrast medium will be administered to confirm sufficient coronary sinus occlusion. The total volume of 60 ml of BMAC preparation, divided into 10 ml injection vials, will be infused into the coronary sinus during over a five-minute period. Subsequently, the balloon catheter will remain inflated for 10 min, to allow cells to migrate to cardiac tissue. After this procedure, patients will be hospitalised for a minimum of 24 h, with ECG and vital signs monitoring.

### Further follow-up

The follow-up visits will be performed according to the protocol after 1, 3, 6 and 12 months (Visits 3, 4, 5 and 6) **[**Table 2**]**, and a physical examination as well as 12 lead ECG and laboratory tests (blood count + diff, clinical biochemistry, troponin I and NT-pro-BNP) will be performed during each visit and any AEs (cardiovascular death, hospitalisation due to decompensation of HF, acute MI, etc.) recorded. An echocardiographic examination will be performed during screening, and MRI examinations will take place on enrolment (Visit 1) and on the final Visit 6 in month 12. LV parameters and a percentage of total myocardial scar (TMS%) and peri-infarct border zone (PI%; PI = 2–3 SD) will be measured using delated myocardial enhancement assessment [5].

In addition, 24-h Holter ECG monitoring will be performed on enrolment (Visit 1) and after 1 month (Visit 3). Exercise tolerance using a six-minute corridor walk test and QoL (KCCQ-12) will be monitored prior to enrolment (Visit 1) and after 6 (Visit 5) and 12 months (Visit 6) **[**Table 2].

Interventional cardiologists will not be blinded to the assigned therapy, but clinical evaluation, measurement of MRI parameters and data processing will be performed by blinded, independent persons.

### Sample size and statistical analysis

The statistical estimate of the size of the file was based on the data from previous studies in patients with congestive heart failure [6] in which was reached the difference of 13.0 + 12.9 ml in LVESV between study and control groups. α type I error of 5%, and β test strength of 80% were used to determine the required group size of 17 patients per group. When including an expected loss of 10% of patients in the 12-month follow-up (including MRI drop out), the resulting size of our group was 40 patients (20 per arm).

Continuous variables with normal distribution will be presented as mean and standard deviation (SD) and will be compared using Student 2-sample *t* test. Continuous variables with non-normal distribution will be presented as the median and range (minimum–maximum or lower and higher percentile) and will be compared using the nonparametric Mann–Whitney *U* test. Categorical variables will be presented as counts and percentages and will be compared using the χ2 or Fisher exact test. Odds ratios (OR) are expressed with 95% CI. A *p* < 0.05 will be considered significant. All statistical analyses will be performed using IBM SPSS Statistics version 22.

## Discussion

Currently, the three primary mechanisms involved in activity of transplanted bone marrow stem cells (BMC) are considered as cardiomyogenesis, vasculogenesis and paracrine effect, the latter of them play a key role in myocardial regeneration. The paracrine effect of BMSC may lead to myocardial protection and neovascularisation and may also influence the inflammation, fibrosis, myocardial contractility, metabolism and the endogenous regeneration of residual cardiomyocytes. The immediate cytoprotective effect of BMC is established by the release of cytoprotective molecules, such as vascular endothelial growth factor (VEGF), platelet-derived growth factor (PDGF) and interleukin-1β (IL-1β) and insulin-like growth factor (IGF-1), leading in vivo to the inhibition of apoptosis and a reduction in infarct size. BMC produces a number of proangiogenic factors, such as VEGF, basic fibroblast growth factor (bFGF), hepatocyte growth factor (HGF), angiopoietin, IL-1β and tumour necrosis factor (TNF-α), that may increase a neovasculogenesis and microvascular density in ischemic tissue. BMC also releases a number of factors accompanied by a paracrine antifibrotic effect affecting myocardial fibrosis and remodelling. Fibroblast proliferation and collagen I and III synthesis are reduced. The expression of tissue inhibitor of metalloproteinase (TIMP-1) and the transforming growth factor (TGF), which affects favourably the extracellular matrix, is increased. The inhibition of negative myocardial remodelling leads subsequently to a reduction in end-diastolic left ventricular pressure and a dP/dt max increase. The paracrine effect of BMC results in a reduction of β-receptor down-regulation in the failing myocardium and thus improves the contractility of residual cardiomyocytes as well as the metabolism of ischemic cardiomyocytes. BMC can affect cardiomyocyte regeneration either directly, through transdiferentiation, or by fusion with native cardiomyocytes, but mainly due to paracrine action (HGF, IGF-1), leading to the mobilisation, proliferation and differentiation of residual endogenous myocardial progenitor cells [7–9].

Platelets are intensively involved in neoangiogenesis, not only alongside haemostasis, but also through intense contact with endothelial cells. Platelets affect the formation of new vessels by releasing large amounts of growth factors such as VEGF, beta-FGF, epidermal growth factor (EGF), platelet-derived growth factor (PDGF) and matrix metalloproteinases (MMPs), but they also contain a large number of inhibitors (e.g. endostatin, platelet factor 4 (PF4), thrombospodin-L, α-2-macroglobulin, plasminogen activator inhibitor [PAI-I] and angiostatin) [7–9].

The main methods involved in applying stem cells to ischemic myocardium are direct intramyocardial injection and intracoronary or retrograde administration via the coronary sinus [10].

Direct intramyocardial stem cell administration leads to high myocardial capture without affecting coronary flow. Percutaneous endoventricular intramyocardial administration using dedicated catheters is relatively safe, although it may be associated with a risk of perforation and tamponade or the induction of ventricular arrhythmias within the first 2 weeks after acute MI. Intramyocardial cell retention may be up to 20–30%, with the remaining cells being retained in pulmonary circulation and the liver. Another advantage of endoventricular administration is the real-time assessment of myocardial viability and contractility, using electromechanical mapping (e.g. NOGA system), and the possibility of targeted stem cell administration to hibernated myocardial areas [9].

Perioperative epimyocardial stem cell injection during CABG allows for targeted administration with visual control, though one disadvantage is the need for surgical access. As an alternative, endoscopic procedures are being developed to ensure transepicardial administration [11].

Intracoronary infusion is the most common route of administration in acute MI patients. The main advantage of this approach is the safety and simplicity of administering stem cells selectively into the infarction artery supplying the ischemised myocardium. However, intracoronary application results in only 1–3% of the cells being captured in the myocardium, with most of the cells being flushed and trapped in the lungs and the liver. Furthermore, infusion of these relatively large cells may lead to the obstruction of coronary microcirculation [11, 12]. Gyöngyösi et al. have shown that absolute myocardial flow after intracoronary stem cell injection decreases immediately by 30%, and this reduction persists for 24 h [13].

Retrograde infusion through the coronary sinus after previous balloon catheter obstruction is an alternative to antegrade intracoronary administration. Retrograde cannulation of the coronary venous system seems to be safe and is commonly used by arrhythmologists, e.g. for implanting the left ventricular pacing leads in resynchronisation therapy [10, 14] or for retrograde cardioplegia during cardiac surgery. This access is reported to be safe, with injury to the CS occurring in 0.06 to 0.6% of patients resulting in formation of hematoma on the atrioventricular groove, perforation of the CS wall, pericardial effusion, or laceration of the right ventricle [15].

In the metaanalysis by Gathier et al., only a transient increase in troponin-I levels was seen after retrograde stem cells infusion with resolution within 24 h. No arrhythmias were detected in this patients and there was no evidence of damage to the CS or increase of mortality rate releated to retrograde CS infusion [15].

However, there is a lack of information about retrograde application of BMSC through coronary sinus in patients with cardiac resynchronisation therapy (CRT) and LV lead in CS. Due to safety reason, these patients will not be included in the study.

Suzuki et al. on a mouse model demonstrated that retrograde infusion through the coronary sinus allows the penetration of cells into single layers of myocardium, with minimal risk of damage. The retention of cells administered in this way is somewhat higher, and in some studies it reaches 3.2% compared to 2.6% in anterograde intracoronary administration [16].

Zhou et al. on a porcine model analysed the tissue distribution of radioactive BMC in various application methods – direct intramyocardial, intra-coronary and retrograde transvenous routes to the coronary sinus. Each of these methods was associated with significant extracellular entrapment of the administered cells in pulmonary circulation (26 +/− 3% vs. 47 +/− 1% and 43 +/− 3%, respectively). The significant majority of the cells were captured in the myocardium in direct intramyocardial administration compared to intra-coronary or retrograde transvenous administration (11 +/− 3% vs. 2.6 +/− 0.3% or 3.2 +/− 1%; *p* = 0.05, respectively), while the difference between the last two was not significant [17].

Silva et al. found that the retention of radiolabelled autologous bone marrow mononuclear cells (BMMNCs) administrated in patients suffering from ST elevation myocardial infarction was higher after anterograde intracoronary delivery compared to the retrograde coronary sinus approach (10.29+/− 6.38% vs. 3.13 +/− 0.99%; *p* = 0.03). Anterograde intracoronary administration was also associated with a significant increase in LV EF (*p* = 0.02) at 6 months [18].

In the REVIVE trial, retrograde BMAC administration into the coronary sinus, in patients with either ischemic (IHF) or non-ischemic HF (IHF), was associated with a significant improvement to LV EF (NIHF: from 25.1 to 31.1%; *p* = 0.007 and IHF: from 26.3 to 31.1%; *p* = 0.035) at the 12-month follow-up. However, a significant decrease in LVESd was observed, albeit only in the NIHF group (from 55.6 to 50.9 mm; *p* = 0.020) [4].

In the study by Vicario et al., retrograde administration of BMAC via the coronary sinus in patients with refractory angina led to a significant reduction in rest and stress-induced ischemic perfusion abnormalities (Tc SPECT) of 40.9% (*p*<0.01) and 45.3% (*p*<0.05), respectively. Using the Seattle Angina Questionnaire (SAQ), an average improvement of 30% in the quality of life was found, and the CCS angina class improved from 3.0+/− 0.55 to 1.6+/− 0.63 (*p*<0.001) after 1 year [19, 20].

The RESCUE-HF Trial found that retrograde delivery of allogeneic umbilical cord subepithelial cells in patients with HF led to significant improvement of LV EF from 25.4+/− 5.5% to 34.9+/− 4.1% [*p* < 0.05] and significant decrease in LVESd from 59.9 +/− 5.3 mm to 52.6 +/− 2.7 mm (*p* < 0.05) at 12 months [21].

The TAC-HFT randomised trial compared the safety and efficacy of transendocardial stem cell injection with autologous mesenchymal stem cells (MSCs) and whole bone marrow mononuclear cells (BMCs) vs. placebo in patients with ischemic HF. In this study, infarct size as a percentage of LV mass was reduced by MSCs (− 18.9%; 95%CI: -30.4 to − 7.4; *p* = 0.004) but not by BMCs (− 7.0%; 95%CI: -15.7-1.7%; *p* = 0 .11), and regional myocardial function as a peak circumferential strain at the site of injection improved with MSCs (− 4.9; 95%CI: -13.3-3.5; *p* = 0.03) but not with BMCs (− 2.1; 95%CI: -5.5-1.3; *p* = 0.21). There were no significant changes in LV volumes or EF. The Minnesota Living with Heart Failure score improved with MSCs (*p* = 0.02) and BMCs (*p* = 0.005) in the 12-month follow-up, but 6-min walk distance increased with MSCs only (*p* = 0.03) [22].

Tuma et al. evaluated the safety and efficacy of autologous bone marrow-derived mononuclear cells (BMMCs) delivered retrograde via coronary sinus perfusion in the treatment of chronic refractory angina pectoris. They found a significant reduction in the area of ischemic myocardium by SPECT, from 38.2 to 23.5% (*p* = 0.001), and an improvement in the LV EF, from 31.2 to 35.5% (*p* = 0.019) at a two-year follow up [23].

In metaanalysis by Afzal et al., BMC transplantation improved LV EF (2.92%; 95% CI: 1.91–3.92; *p* < 0.00001), reduced infarct size (− 2.25%; 95%CI: − 3.55 to − 0.95; *p* = 0.0007) and LVESV (− 6.37 mL; 95%CI: − 8.95 to − 3.80; *p* < 0.00001), and tended to reduce LVEDV (− 2.26 mL; 95%CI: − 4.59 to 0.07; *p* = 0.06) compared with standard therapy. During follow-up, BMC therapy was also associated with significantly lower incidence of all-cause mortality (OR 0.55; 95%CI: 0.34–0.89; *I*^*2*^ = 37%; *p* = 0.01), recurrent MI (OR: 0.50; 95%CI: 0.27–0.92; *I*^*2*^ = 21%; *p* = 0.03), ventricular tachycardia/fibrillation (OR: 0.45; 95%CI: 0.22–0.93; *I*^*2*^ = 10%; *p* = 0.03), and cerebrovascular accidents (OR: 0.25; 95%CI: 0.08–0.81; *I*^*2*^ = 0%; *p* = 0.02) [24, 25].

Like any other cell in the body, stem cells endure chronological aging. Aging of stem cells is generally contemplated as multifactorial and encompasses various contributory mechanisms (e.g. accumulation of reactive oxygen species, metabolic changes, proteostasis, telomere shortening, DNA damage and mutations, niche damage and epigenetic alterations etc.) [26].

Senderup et al. compared the proliferative capacity of bone marrow stromal cell (MSC) cultures from young (aged 18–29 years) and old (aged 68–81 years) donors. MSCs from old donors exhibited a decreased maximal life span compared with cells from young donors (24 +/− 11 population doublings [PD] vs. 41+/− 10 PD, *p*<0.05), and the mean PD rate was lower in old donor cells (0.05 +/− 0.02 PD/day) compared with young donor cells (0.09+/− 0.02 PD/day) [*p*<0.05]. No differences were detected in the numbers of senescence-associated β-galactosidase-positive (SA β–gal+) cells or the mean telomere length in early passage cells obtained from young and old donors. However, MSCs from old donors exhibited accelerated senescence, evidenced by the increased numbers of SA β–gal+ cells per PD compared with the young participants (4% per PD vs. 0.4% per PD, respectively) [27].

A direct comparison of young and old BMSCs from human donors (age 17–90 years) showed age-related multifold increase in SA β–gal+ cells in the old BMSCs, which had nearly twofold longer doubling time in culture [28].

It seems that age of the donors cells remains an important factor to consider in the clinical studies. Therefore, our study will include patients aged 40–80 years.

Harvest BMAC concentrate differs from the concentrate prepared using the Ficoll method, which is considered standard. Ficoll concentrate primarily contains mononuclear cells (60+/− 20%) and only 5% of granulocytes and 0.5% of platelets. In addition, the Ficoll method requires manual steps to stretch the plasma, platelet layers and mononuclear layers before washing out any additional material from the separated cells. These steps depend on the operator and must be performed in a sterile laboratory environment [29].

The Harvest BMAC system concentrates the entire population of nuclear cells from bone marrow aspirates together with platelets. This technique is much faster and does not depend on the operator, so it is less prone to any human factor failure. BMAC concentrate contains mononuclear cells, thrombocytes, circulating endothelial progenitors of CD34+/CD31- and CFU-fibroblasts. The presence of both platelets and additional granulocytes can have a positive effect on the neovascularisation potential of the resulting concentrate – an effect attributed to the increased presence of “angiogenic factors” (especially VEGF) and cytokines [29]. The cellular composition of BMAC and growth factors release following clotting by autologous thrombin is presented in Table 3 [29].

**Table 3:** The cellular composition of BMAC and growth factors concentration

Total nucleated cells (×10^6^/ml)	89.1 ± 8
Total mononuclear cells (×10^6^/ml)	18.80 ± 3.41
CD34+ cells (×10^3^/ml)	800 ± 180
Platelet count (×10^3^/μl)	752 ± 509
PDGF-AB (ng/ml)	752 ± 509
PDGF-AB (ng/ml)	86.8 ± 80.1
TGF-β1 (ng/ml)	124.6 ± 70.2

In order to assess the clinical effect of the therapy, patients enrolled in a study should be in a stable condition. Heart failure may be assessed as chronic at least 6 month after a myocardial infarction (MI), and before enrolling in the study, patients must have been on standard HF therapy for at least for 3 months (ACE inhibitor/ATII blockers, beta-blockers, MRA [with titration to the maximum tolerated dose] and diuretics) and in a stabilised state for at least 1 month (no hospitalisation for HF, stable NYHA class, no diuretic dose variation) [30].

Several limitations relating to blinding should be mentioned. Due to ethical reasons, patients randomised to the control group will not undergo bone marrow collection. However, the intervention will be blinded, and a right heart catheterisation as a sham procedure will be performed in this group. Interventional cardiologists will not be blinded to the assigned therapy, but clinical evaluation, measurement of MRI parameters and data processing will be performed by blinded, independent persons.

## Conclusion

Retrograde administration on non-selected BMAC via coronary sinus, due to the content of platelets and growth factors, might improve observed left ventricular function parameters and improve exercise tolerance in patients with heart failure caused by the systolic dysfunction of ischemic aetiology.

## Abbreviations

ACD-A: Anticoagulant citrate dextrose solution A
ACE: Angiotensin-converting enzyme
AE: Adverse event
ALT: Alanine aminotransferase enzyme
ASA: Acetylsalicylic acid
AST: Aspartate aminotransferase enzyme
AT II: Angiotensin II
ATB: Antibiotics
bFGF: Basic fibroblast growth factor
BMAC: Bone marrow autologous cells concentrate
BMC: Bone marrow stem cells
BMI: Body mass index
BMMNC: Bone marrow mononuclear cells
CABG: Coronary artery bypass graft
CAD: Coronary artery disease
CCS: Canadian cardiovascular society classification of angina pectoris
CK-MB: MB isoenzyme of creatine kinase
CRT: Cardiac resynchronization therapy
ECG: Electrocardiography
ECHO: Echocardiography
EGF: Epidermal growth factor
G- CSF: Granulocyte-colony stimulating factor
HF: Heart failure
HFREF: Heart failure with reduced ejection fraction
HGF: Hepatocyte growth factor
HTC: Hematcrit
ICD: Implantable cardioverter-defibrilator
IGF − 1: Insulin-like growth factor − 1
IL-1β: Interleukin-1β
KCCQ-12: Kanas City Cardiomyopathy Questionare
LAD: Left anterior descending artery
LCx: Left circumflex artery
LV EF: Left ventricular ejection fraction
LVESd/EDd: Left ventricular end-systolic/end-diastolic diameter
LVESV/EDV: Left ventricular end-systolic/end-diastolic volume
MI: Myocardial infarction
MMP: Matrix metalloproteinase
MRA: Mineralocorticoid receptor antagonist
MRI: Magnetic resonance imaging
MSC: Mesenchymal stem cells
NOGA: Electroanatomical mapping system
NT-pro-BNP: N-terminal prohormone of brain natriuretic peptide
NYHA: New York Heart Association functional classification
OR: Odds ratio
PAI-I: Plasminogen activator inhibitor-1
PDGF: Platelet-derived growth factor
PI%: Percentage of peri-infarct border zone
QoL: Quality of life
SD: Standard deviation
SPECT: Single-photon emission computed tomography
TGF: Transforming growth factor
TIMP-1: Tissue inhibitor of metalloproteinase
TMS%: Percentage of total myocardial scar
TNF-α: Tumour necrosis factor
TVR: Target vessel revascularisation
ULN: Upper limit of normal
VEGF: Vascular endothelial growth factor

## References

[1] Jeevanantham V, Butler M, Saad A, Abdel-Latif A, Zuba-Surma EK, Dawn B (2012). Adult bone marrow cell therapy improves survival and induces long-term improvement in cardiac parameters a systematic review and meta-analysis. Circulation.

[2] van der Spoel TIG, Lorkeers SJ, Agostoni P, van Belle E, Gyongyosi M, Sluijter JPG (2011). Human relevance of pre-clinical studies in stem cell therapy: systematic review and meta-analysis of large animal models of ischaemic heart disease. Cardiovasc Res.

[3] Wu KH, Hanb ZC, Moa XM, Zhou B (2012). Cell delivery in cardiac regenerative therapy. Ageing Res Rev.

[4] Patel AN, Mittal S, Turan G, Winters AA, Henry TH, Ince H (2015). REVIVE trial: retrograde delivery of autologous bone marrow in patients with heart failure. Stem Cells Transl Med.

[5] Kwon DH, Asamoto L, Popovic ZB, Kusunose K, Robinson M, Desai M (2014). Infarct characterization and quantifcation by delayed enhancement cardiac magnetic resonance imaging is a powerful independent and incremental predictor of mortality in patients with advanced ischemic cardiomyopathy. Circ Cardiovasc Imaging.

[6] Mathiasen AB, Qayyum AA, Jørgensen E, Helqvist S, Fischer-Nielsen A, Kofoed KF (2015). Bone marrow-derived mesenchymal stromal cell treatment in patients with severe ischaemic heart failure: a randomized placebo-controlled trial (MSC-HF trial). Eur Heart J.

[7] Lerman DA, Alotti N, Ume KL, Péault B (2016). Cardiac Repair And Regeneration: The Value Of Cell Therapies. Eur Cardiol Rev.

[8] Katarzyna R (2017). Adult stem cell therapy for cardiac repair in patients after acute myocardial infarction leading to ischemic heart failure: an overview of evidence from the recent clinical trials. Curr Cardiol Rev.

[9] Takashima S, Tempel D, Duckers HJ. Current outlook of cardiac stem cell therapy towards a clinical application. Heart 2013;0:1–13. doi:10.1136/heartjnl-2012-303308. 10.1136/heartjnl-2012-303308 23525708

[10] Sheng CC, Zhou L, Hao J. Current Stem Cell Delivery Methods for Myocardial Repair. BioMed Res Int*.* 2013; Article ID 547902: 1–15. 10.1155/2013/547902 10.1155/2013/547902 PMC3591183 23509740

[11] Strauer BE, Steinhoff G (2011). 10 Years of Intracoronary and Intramyocardial Bone Marrow Stem Cell Therapy of the Heart From the Methodological Origin to Clinical Practice. JACC.

[12] Delewi R, Andriessen A, Tijssen JGP, Schächinger V, Wojakowski W, Roncalli J (2013). Impact of intracoronary cell therapy on left ventricular function in the setting of acute myocardial infarction: a meta-analysis of randomised controlled clinical trials. Heart.

[13] Gyöngyösi M, Giurgea GA, Syeda B, Charwat S, Marzluf B, Mascherbauer J (2016). Long-term outcome of combined (Percutaneous intramyocardial and intracoronary) application of autologous bone marrow mononuclear cells post myocardial infarction: the 5-year MYSTAR study. PLoS One.

[14] Wu K, Mo X, Lu S, Han Z (2011). Retrograde delivery of stem cells: promising delivery strategy for myocardial regenerative therapy. Clin Transpl.

[15] Gathier WA, van Ginkel DJ, van der Naald M, van Slochteren FJ, Doevendans PA, Chamuleau SAJ (2018). Retrograde coronary venous infusion as a delivery strategy in regenerative cardiac therapy: an overview of preclinical and clinical data. J Cardiovasc Transl Res.

[16] Suzuki K, Murtuza B, Fukushima S, Smolenski RT, Varela-Carver A, Coppen SR (2004). Targeted cell delivery into infarcted rat hearts by retrograde intracoronary infusion: distribution, dynamics, and influence on cardiac function. Circulation.

[17] Zhou R, Acton PD, Victor A, Ferrari VA (2006). Imaging stem cells implanted in infarcted myocardium. J Am Coll Cardiol.

[18] Silva SA, Sousa ALS, Haddad AF, Azevedo JC, Soares VE, Peixoto CM (2009). Autologous bone-marrow mononuclear cell transplantation after acute myocardial infarction: comparison of two delivery techniques. Cell Transplant.

[19] Vicario J, Campos C, Piva J, Faccio F, Gerardo L, Becker C (2004). Transcoronary sinus administration of autologous bone marrow in patients with chronic refractory stable angina phase 1. Cardiovasc Radiat Med.

[20] Ortega H, Pierini A, Lofeudo C, Novero R, Balino NP, Monti A (2005). One-year follow-up of transcoronary sinus administration of autologous bone marrow in patients with chronic refractory angina. Cardiovasc Revasc Med.

[21] Tuma J, Carrasco A, Castillo J, Cruz C, Carrillo A, Ercilla J (2016). RESCUE-HF trial: retrograde delivery of allogeneic umbilical cord lining subepithelial cells in patients with heart failure. Cell Transplant.

[22] Heldman AW, DiFede DL, Fishman JE, Zambrano JP, Trachtenberg BH, Karantalis V (2014). Transendocardial mesenchymal stem cells and mononuclear bone marrow cells for ischemic cardiomyopathy: the TAC-HFT randomized trial. JAMA.

[23] Tuma J, Fernández-Viña R, Carrasco A, Castillo J, Cruz C, Carrillo A (2011). Safety and feasibility of percutaneous retrograde coronary sinus delivery of autologous bone marrow mononuclear cell transplantation in patients with chronic refractory angina. J Transl Med.

[24] Afzal MR, Samanta A, Shah ZI, Jeevanantham V, Abdel-Latif A (2015). Adult bone marrow cell therapy for ischemic heart disease evidence and insights from randomized controlled trials. Circ Res.

[25] Shahid MSS, Lasheen W, Haider KH (2016). The modest outcome of clinical trials with bone marrow cells for myocardial repair: is the autologous source of cells the prime culprit?. J Thorac Dis.

[26] Haider KH (2018). Bone marrow cell therapy and cardiac reparability: better cell characterization will enhance clinical success. Regen Med.

[27] Stenderup K, Justesen J, Clausen C, Kassem M (2003). Aging is associated with decreased maximal life span and accelerated senescence of bone marrow stromal cells. Bone.

[28] Zhou S, Greenberger JS, Epperly MW, Goff JP, Adler C, Leboff MS (2008). Age-related intrinsic changes in human bone marrow-derived mesenchymal stem cells and their differentiation to osteoblasts. Aging Cell.

[29] Hermann PC, Huber SL, Herrler T, von Hesler C, Andrassy J, Kevy SV (2008). Concentration of bone marrow total nucleated cells by a point-of-care device provides a high yield and preserves their functional activity. Cell Transplant.

[30] Ponikowski P, Voors AA, Anker SD, Bueno H, Cleland JGF, Coats AJS (2016). 2016 ESC guidelines for the diagnosis and treatment of acute and chronic heart failure the task force for the diagnosis and treatment of acute and chronic heart failure of the European Society of Cardiology (ESC) developed with the special contribution of the heart failure association (HFA) of the ESC. Europ Heart J.

